# The Current Status of Somatostatin-Interneurons in Inhibitory Control of Brain Function and Plasticity

**DOI:** 10.1155/2016/8723623

**Published:** 2016-06-14

**Authors:** Isabelle Scheyltjens, Lutgarde Arckens

**Affiliations:** Laboratory of Neuroplasticity and Neuroproteomics, KU Leuven, 3000 Leuven, Belgium

## Abstract

The mammalian neocortex contains many distinct inhibitory neuronal populations to balance excitatory neurotransmission. A correct excitation/inhibition equilibrium is crucial for normal brain development, functioning, and controlling lifelong cortical plasticity. Knowledge about how the inhibitory network contributes to brain plasticity however remains incomplete. Somatostatin- (SST-) interneurons constitute a large neocortical subpopulation of interneurons, next to parvalbumin- (PV-) and vasoactive intestinal peptide- (VIP-) interneurons. Unlike the extensively studied PV-interneurons, acknowledged as key components in guiding ocular dominance plasticity, the contribution of SST-interneurons is less understood. Nevertheless, SST-interneurons are ideally situated within cortical networks to integrate unimodal or cross-modal sensory information processing and therefore likely to be important mediators of experience-dependent plasticity. The lack of knowledge on SST-interneurons partially relates to the wide variety of distinct subpopulations present in the sensory neocortex. This review informs on those SST-subpopulations hitherto described based on anatomical, molecular, or electrophysiological characteristics and whose functional roles can be attributed based on specific cortical wiring patterns. A possible role for these subpopulations in experience-dependent plasticity will be discussed, emphasizing on learning-induced plasticity and on unimodal and cross-modal plasticity upon sensory loss. This knowledge will ultimately contribute to guide brain plasticity into well-defined directions to restore sensory function and promote lifelong learning.

## 1. Introduction

The dynamic and delicate interplay of excitatory and inhibitory neurons allows the brain to process, adapt, and respond to environmental cues coming in through the sensory systems. In the healthy brain, inhibitory transmission in general prevents runaway excitation and sharpens the response properties of excitatory neurons [1, 2]. By putting a brake on cortical excitability, inhibitory neurons provide temporal precision to cortical firing, enhance the saliency of sensory inputs, and promote long-range synchrony [3, 4]. Looking at absolute numbers, inhibitory cells constitute only a minority of cortical neurons (20%) compared to the abundant population of excitatory cells (80%), yet inhibitory neurons display a much richer diversity [5]. The exact number of subpopulations is ever increasing as new markers are continuously discovered, keeping classification an ongoing matter of debate [6–10]. Inhibitory subpopulations can be divided, not only based on molecular fingerprints, but also based on differences in physiological and synaptic properties and their diverse dendritic and axonal morphologies [2, 11–14], underscoring functionally distinct roles in mediating cortical processing. Only now, with the emergence of powerful and specific neuroscience tools, are we beginning to comprehend the diverse functionality of inhibitory neurotransmission in maintaining stability and control over brain development and function. The search for interneuron function becomes crucially important in pathological conditions, as an imbalance between excitation and inhibition is associated with a wide range of neurological disorders ranging from autism to schizophrenia, depression, and epilepsy [3, 15], or the cognitive deficits associated with the syndromes of Down and Rett [16]. In case a sensory system fails, as a result of deprivation or deafferentation, a compensatory plastic reorganization of the affected sensory cortex will take place, bestowing the brain the remarkable ability to reinforce the remaining modalities and to reallocate the sensory-deprived cortical areas to different functions. In this context as well, inhibition is a paramount player in directing and restricting brain plasticity, not only during sensitive periods in the course of brain development [17], but also throughout adulthood [18]. On the downside, plasticity can also result in pathological conditions or can cause maladaptive brain reorganization [19]. Impaired or excessive plasticity, often linked to an excitation-inhibition imbalance, can severely hamper normal brain functioning to the extent of causing cognitive disabilities [20–22]. Guiding plasticity into well-defined directions can therefore lead to more effective therapies to improve or restore function, to invigorate lifelong learning, or to allow better processing of sensory implants as a cure for sensory deficits [23]. The exact molecular and cellular underpinnings of plasticity mechanisms however and especially how distinct subsets of inhibitory neurons contribute to these phenomena remain unclear. Innovative neuroscience and therapeutic tools allow us to specifically target and modulate well-defined neuronal subsets. Most of these tools use molecular markers to target cell classes, and generally, inhibitory neurons can be divided into three large, nonoverlapping populations that together constitute almost 100% of inhibitory neocortical neurons: parvalbumin- (PV-), somatostatin- (SST-), and serotonin receptor 3a- (5HT_3a_R-) expressing inhibitory neurons [24]. A vast amount of work has focused on PV-interneurons in experience-dependent plasticity, more precisely in controlling developmental windows for ocular dominance plasticity (for a review see [25]). In contrast, the potential roles of SST- and 5HT_3a_R-expressing populations in both normal cortical processing and plasticity are far less understood. Nevertheless, SST-interneurons are promising candidates in the context of mediating experience-dependent cortical plasticity through inhibition of distal dendrites of pyramidal neurons where intracortical inputs converge. As such they are ideally positioned to control plasticity of excitatory inputs that synapse onto the same dendritic location. At the same time, they strongly innervate PV-interneurons and are as such well-positioned to regulate inhibition instructive to brain plasticity [26–28]. A confounding factor in investigating interneuron function however is that these three general inhibitory subclasses are highly diverse themselves [29], including the SST-interneurons. As many studies today use transgenic mouse lines to investigate interneuron function, it is sometimes unclear which subpopulation is targeted, or whether multiple SST-subpopulations are targeted at the same time [30], which would preclude straightforward interpretations of the datasets. Therefore this review will commence with a general introduction into interneurons with an emphasis on mouse neocortex, to then explain in more detail the subdivision and specific functions in cortical processing of the SST-interneurons known so far, before looking into how these SST-interneurons may mediate specific cortical plasticity phenomena.

## 2. Interneurons in the Mammalian Neocortex

Inhibitory neurons use gamma-amino butyric acid (GABA) as their main inhibitory neurotransmitter and are mostly local-circuit interneurons. Their axonal arborization is typically restricted to the neocortex and does not project into the white matter [13]. Nonetheless, small populations of long-distance projecting GABAergic neurons have been described [31, 32]. Transcallosal GABAergic neurons have been reported in motor cortex [33], somatosensory cortex [34], and visual cortex [35], but also ipsilateral projecting GABAergic neurons have been described [36–40]. These projecting neurons make up 0.5% of the total amount of inhibitory cortical neurons and their function is still poorly understood [41]. Therefore, this review will mainly focus on local-targeting GABAergic neurons, which will be further referred to as interneurons.

In general, most attempts to classify interneurons are based on the expression of different molecular markers, morphological and electrophysiological properties [8, 9]. Recently, large-scale single-cell RNA sequencing has revealed at least 16 molecularly distinct classes of interneurons, 14 of which are present in the mouse neocortex [42]. This is the same number as the 14 electrophysiological classes described by Gupta et al. [8], but a clear correlation between molecular, morphological, and electrophysiological properties to classify interneurons in a straightforward way is missing, making it very difficult to compare subsets of interneurons described in different studies. The molecular markers based on which GABAergic neurons are generally divided are the calcium-binding proteins parvalbumin (PV), calbindin (CB), and calretinin (CR), the neuropeptides somatostatin (SST), neuropeptide Y (NPY), cholecystokinin (CCK), and vasoactive intestinal peptide (VIP), the ionotropic serotonin receptor 5HT_3a_R, and neuronal nitric oxide synthase (nNOS). Sets of these molecular markers partially overlap to different extents in distinct subtypes [7], but the general consensus is that PV-, SST-, and 5HT_3a_R-interneurons make up three nonoverlapping classes, together forming approximately 100% of the interneurons in mouse cortex [5, 24, 42–44]. Most PV-interneurons (40% of neocortical interneurons) have either a basket cell or Chandelier cell morphology. Basket cells target somata and proximal dendrites of pyramidal neurons and have been mainly described in supragranular layers. Chandelier cells exhibit extensive axonal branching and their terminals mainly target the distal regions of axon initial segments of pyramidal cells. These cells are found in layers II–VI. Electrophysiologically, both cell types are fast-spiking [45]. A well-known population of SST-interneurons (30% of neocortical interneurons) consists of the layer I dendrite-targeting Martinotti cells, of which the spindle shaped cell bodies are found in layers II/III and V and less frequently in layer VI. These are generally low-threshold regularly spiking neurons [46]. 5HT_3a_R-interneurons (30% of neocortical interneurons) can be largely subdivided into VIP- and non-VIP-expressing interneurons [43, 47–49]. VIP-interneurons are either Double-bouquet cells, bipolar cells, or bi-tufted cells and are mostly found in supragranular layers, although they have also been described in layers V (for Double-bouquet cells) and VI (for bipolar and bi-tufted cells). Double-bouquet cells have ovoid somata and are dendrite-targeting as they innervate dendritic spines and shafts. Electrophysiologically they are characterized as non-fast-spiking cells. Bipolar cells have a fusiform somatodendritic arborization, with two main opposing, vertically oriented dendrites. Their firing pattern ranges from regular to irregular spiking, with an initial bursting response followed by accommodating spikes [43]. Bi-tufted cells also have ovoid somata and vertically oriented bi-tufted dendrites, but with a wider horizontal axonal span and a less extensive vertical projection compared to Double-bouquet or bipolar cells. They are also mainly dendrite-targeting cells [12]. Non-VIP-5HT_3a_R-interneurons are mostly small neurogliaform cells with a large number of short, radiating dendrites forming spherical structures and have highly branched, thin axons [50]. Neurogliaform cells have been described in all cortical layers and belong to the category of late-spiking cells [12, 45] (for more detailed information about inhibitory subclasses see the following reviews: [2, 12, 13, 51]).

Further subdividing interneurons based solely on molecular markers has its limitations as distinct functional subclasses can express the same calcium-binding proteins or neuropeptides, and this may be functionally not very informative. On the other hand, many studies currently take advantage of the availability of many transgenic mouse lines in which molecular interneuron subclasses can be reliably and reproducibly targeted across experiments and laboratories in order to elucidate their functional roles in cortical processing [52, 53]. As such, several of these mouse lines have aided in the discovery of multiple distinct subtypes of SST-interneurons in addition to the well-described set of Martinotti cells [54–56].

## 3. Subdivision of SST-Positive Inhibitory Neurons

**Figure 1:**
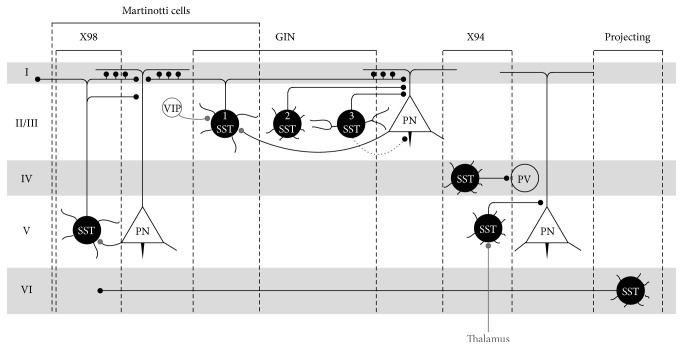
SST-interneurons labeled in X98-, GIN-, and X94-transgenic mice have distinct laminar distributions and wiring patterns. X98-SST-interneurons mainly reside in infragranular layer V whereas the GIN-SST-interneuron subpopulation 1 mainly resides in supragranular layer II/III. Both subtypes are considered Martinotti cells due to their layer I dendrite-targeting properties onto layer II/III and V pyramidal neurons. The second and third population of GIN-SST-interneurons avoid layer I but dendritically target pyramidal neurons within layer II/III. GIN-type 2 interneurons are characterized by small, multipolar dendritic arbors, whereas GIN-type 3 interneurons have larger, bitufted dendritic arbors. Some layer II/III GIN-SST-neurons target the axon initial segment of pyramidal neurons. The dotted line denoting this synapse indicates that it is not yet known to which subpopulations this property can be attributed. Layer II/III VIP-interneurons somatically target SST-interneurons within this layer. X94-SST-interneurons reside either in layer IV, where they mainly target fast-spiking PV-interneurons, or in layer V, where they dendritically target layer V pyramidal neurons. Specifically layer V X94-neurons can receive thalamic input, whereas layer IV X94-neurons are intracortically driven. Finally, SST-projecting-neurons are mainly described in layer VI. Thick lines indicate dendritic arbors; thin lines depict axonal projections. Black dots indicate synapses between SST-interneuron dendrites and their targets. Grey dots indicate synapses from the input sources onto SST-interneurons.

It has been clear for some time that the cortical SST-inhibitory cell population is not homogeneous. Several distinct SST-populations, in addition to Martinotti cells, have already been described in the mouse cortex, and more are likely to follow [54–57]. These observations are based on distinct electrophysiological, anatomical [58], and molecular properties [44]. This review will first discuss in more detail the characteristics of Martinotti cells before comparing their properties with other SST-subsets in order to get a better understanding of the distinct functional features in cortical processing and plasticity mechanisms.  Figure 1 shows a schematic overview of the distinct SST-subpopulations so far described and their most important input- and output relationships, which will be further discussed in the following sections.

### 3.1. Martinotti Cells

Martinotti cells, described for the first time in 1889 by Martinotti [59], constitute the best-known and largest SST-interneuron subpopulation. Approximately 15% of the neocortical interneurons and 50% of the SST-interneurons are Martinotti cells [13, 60]. Anatomically, their somata are located mostly in layers II/III and V and to a lesser extent in layer VI [55]. Martinotti cell somata are spindle or ovoid shaped and their dendrites are locally extensively arborized in a bi-tufted or multipolar fashion, reaching a diameter of 300 *μ*m [61]. A characteristic feature of all Martinotti cells is their long, translaminar ascending axon collaterals with spine-like boutons reaching layer I, where they branch out and spread horizontally up till 2000 *μ*m [62]. Within this layer, their main targets are distal dendritic tufts of pyramidal cells, of which the cell bodies largely reside in layer V [27, 63, 64]. In addition, Martinotti cells are also known to target apical and basal dendrites of pyramidal neurons and other interneurons, mostly fast-spiking PV-interneurons [26], as well as somata of pyramidal neurons and of interneurons present in layer I (Cajal-Retzius cells) [60]. Physiologically, Martinotti cells are generally considered low-threshold, regular spiking interneurons as suprathreshold firing can be elicited by a single presynaptic pyramidal neuron [13]. This is in contrast to high threshold spiking pyramidal neurons or fast-spiking PV-interneurons where excitation of individual inputs is insufficient to generate suprathreshold activation [65–68]. Nevertheless, the physiological responses of Martinotti cells can vary depending on which layer they are located in [60]. Mostly, at steady state, Martinotti cells respond with bursting type spike frequency adaptation or, in a smaller subset, a nonbursting type of adaptation. Small populations with nonadapting bursting or irregular spiking responses have also been reported [6, 8, 60]. Molecularly, all Martinotti cells express SST, and 50% of these SST-positive Martinotti cells also contain other calcium-binding proteins or neuropeptides such as CB, NPY, or CCK, whereas CR [57], PV, and VIP are never coexpressed [13, 60]. Part of the Martinotti cells contains nNOS. A correlation between nNOS in Martinotti cells and the nNOS receptor, guanylyl cyclase, in the apical dendrites of layer V pyramidal neurons [69], reinforces the notion that layer V pyramidal neurons are a main target of Martinotti cells. Recently, a study has shown that a subset of SST-interneurons overlaps with preprodynorphin (PPD). This study considers Martinotti cells likely candidates to express PPD as they also see no CR-coexpression [70]. In summary, it remains open for discussion whether the distinct electrophysiological and molecular features reflect a larger diversity (i.e., the existence of multiple Martinotti cell subtypes) [8], or variability (i.e., within-group differences) of Martinotti cells [60]. The recent observation of the possible presence of PPD in Martinotti cells could provide new, not yet described functional properties for Martinotti cells in cortical processing. Martinotti cells are known to send axons not only to layer I, but also locally in the vicinity of their cell soma. This may imply that, in addition to inhibiting distal apical dendrites of layer V pyramidal neurons, local release of PPD-derived peptides together with the neuromodulator SST could suppress *κ*-opioid and/or SST-receptor-expressing pyramidal neurons present in layers V-VI in a long-lasting manner [70]. Finally, the bursting phenotype sometimes occurring in Martinotti cells could be used to reset the cortical column after intense output, as pyramidal neurons also mostly show bursting for more intense signal transmission [60].

### 3.2. Distinct SST-Subpopulations: GIN-, X98-, and X94-Mouse Strains

Studies that have looked into the characteristics of Martinotti cells have focused largely on morphological properties to identify the proper subset of interneurons, such as the presence of layer I-targeting axon collaterals [60]. However, not all SST-positive interneurons share this feature, as is evidenced by the use of transgenic mouse lines that express green fluorescent protein (GFP) under control of the GAD67 promoter in SST-interneurons. Three such mouse-strains have been studied in literature and express GFP in distinct subsets of SST-interneurons [54]. The GFP expressing inhibitory neuron- (GIN-) [71], X98-, and X94-lines express GFP in some, yet not all SST-interneurons in hippocampus and cortex. By studying these three lines, Ma et al. [54] showed the existence of at least two distinct SST-subsets based on the combination of morphological, molecular, and electrophysiological criteria. The most pronounced differences were reported between the X98- and X94-GFP SST-interneurons, while X98-interneurons were more similar to most GIN-interneurons, and both shared similar features with Martinotti cells.

#### 3.2.1. GIN- and X98-Interneurons

X98-neurons are found in layers V and VI, GIN-neurons in layers II/III and to a lesser extent in layer V. Both are mainly layer I dendrite-targeting neurons and colocalize with CB, like Martinotti cells [58, 72], and partially with NPY. Also like Martinotti cells, they mostly show low-threshold spiking behavior. These observations suggest that X98-neurons and GIN-neurons are mostly Martinotti cells residing in layers V and II/III, respectively (Figure 1).

#### 3.2.2. X94-Interneurons

On the other hand, X94-neurons were shown to be present in layers IV and V. The layer IV and V SST-interneurons show no similarity with Martinotti cells based on electrophysiological or morphological properties and thus most likely belong to a distinct SST-subpopulation. Almost no axon branches to layer I were found; instead they remain local within layer IV, where they inhibit other fast-spiking interneurons such as PV-interneurons [73] or, in the case of layer V X94-SST-interneurons, dendrites of layer V pyramidal cells [74] (Figure 1). X94-neurons do not colocalize with CB and do not show a low-threshold spiking behavior. Instead they demonstrate a stuttering phenotype and electrophysiological parameters very similar to those of fast-spiking interneurons [54, 73]. Furthermore, even though only half of the SST-interneurons in layer IV are labeled in the X94-line, the other SST-interneurons within this layer share the same electrophysiological and morphological properties, indicating that most, if not all, layer IV SST-interneurons are X94-cells [73].

### 3.3. Distinct SST-Subpopulations within the GIN-Strain

A closer look into the characteristics of GFP-interneurons in the GIN-line revealed the presence of SST-interneurons lacking the typical Martinotti cell morphology and behavior, suggesting a larger heterogeneity in SST-subclasses than originally presumed [55, 56]. In the study of Halabisky et al. [56], an unsupervised cluster analysis based on whole-cell patch-clamp recordings was used to compare electrophysiological variables related to the kinetics of spontaneous excitatory postsynaptic currents, firing behavior, and intrinsic membrane properties of the GFP-expressing SST-interneurons in the sensorimotor cortex. As such, at least four distinct subgroups of SST-interneurons with possibly distinct functional roles were clustered. Additional evidence for the presence of multiple subtypes comes from the molecular observation that 33% of GFP-expressing SST-interneurons colocalize with CB, and 40% with CR. Considering the generally perceived absence of CR in Martinotti cells [57], this further indicates the existence of at least several non-Martinotti cell SST-subpopulations. This study done in slices could however not readily correlate morphological information with the observed electrophysiological properties, thereby possibly over- (or under-) estimating the number of distinct SST-subpopulations that agrees with a morphological classification. They reported at least some GFP-interneurons having extensions to layer I, indicating one of the four subgroups is possibly Martinotti cells [56].

Another study revealed at least three distinct clusters of SST-subtypes in the GIN-strain. Here an unsupervised cluster analysis combined electrophysiological and morphological features and as such verified the presence of Martinotti cell-properties in 50% of GFP-interneurons with extensively branching layer I-targeting axon collaterals (Figure 1, SST-interneuron 1). In addition, they reported the existence of two cell types that show some similarities in spike frequency adaptation with two electrophysiologically distinct subpopulations described previously [56]. Molecular details are missing however, impeding comparisons with other studies. Morphologically, these two new groups (termed groups 2 and 3) both show few ascending axons with very few branching points that avoid layer I, and instead all bended medially at the layer I border. The two groups differ however in their dendritic morphology. Group 2 displays the smallest dendritic arbor in a multipolar morphology, whereas group 3 has a larger dendritic arbor organized in bi-tufted manner. Both groups were found in layers II/III, but also, although less, in IV and V (Figure 1, SST-interneurons 2 and 3). All three morphological groups correlated with three electrophysiological groups, where group 1 also shows Martinotti cell-like behavior: low-threshold, non-fast-spiking interneurons with a moderate frequency adaptation. The second cluster contains cells with more hyperpolarized resting membrane potentials and they show both regular and stuttering firing patterns with greater spike frequency adaptation. The third cluster of cells is also more hyperpolarized and shows regular spiking with a similar degree of frequency adaptation, but more narrow action potentials. To some extent, groups two and three show some similarities with the X94-population described by Ma et al. [54], for example, the avoidance of layer I and the stuttering properties of the second group. However the axons of X94-cells are highly branched and localized within layer IV and show quasi-fast-spiking responses, reinforcing the observation that X94-cells likely do make up a subpopulation that is distinct from GIN-cells [54, 55].

### 3.4. Long-Distance Projecting SST-Inhibitory Neurons

As mentioned previously, long-range GABAergic neurons have also been reported in the cortex. Ipsilateral projecting GABAergic neurons are only considered a small fraction of cortical GABAergic neurons (0.5%), yet the largest subset of these projecting cells is SST-positive (91%) and colocalizes with nNOS and NPY. They have been found mainly in layer VI and the white matter, but also layer II/III, and connect cortical areas across the areal boundaries [36] (Figure 1). Recently, a subset of SST-interneurons has been shown to express Lypd6, a member of the lynx family of modulators of nicotinic acetylcholine receptors (nAChR), which more specifically enhances Ca^2+^-currents through nAChRs [75]. Lypd6 was found in a subset of CB+, NPY+ SST-INs, suggesting these are Martinotti cells [54]. It was also found in long-range corticocortical SST-inhibitory neurons, projecting to S1. This could imply that Lypd6-expressing SST-neurons could form yet another distinct subset with a unique function in modulating cortical processing or rhythmic oscillatory activity through the convergence of GABAergic transmission and nicotinic signaling [76].

### 3.5. Distinct Functional SST-Subtypes Based on Differences in Ion Channels and Calcium-Binding Proteins

Previous studies predicting electrical properties based on single-cell gene expression profiles found that ion channels are clustered around specific calcium-binding proteins, characteristic for particular interneuronal subpopulations. These ion channel clusters likely account for the unique electrophysiological properties present in different interneuron subclasses, and moreover also within the SST-interneuron populations. Several ion channels such as the voltage-gated potassium channels Kv2.1, Kv3.3, Kv4.2, and Kv3.1 and the hyperpolarization activated sodium/potassium channel HCN4, for example, are found in Martinotti cells, but not in fast-spiking interneurons or CB-negative SST-interneurons [77]. In addition, Ca*β*1 and Ca*β*4 (auxiliary subunits of the voltage-activated calcium channel family), HCN3, Ca*α*1G (gives rise to a T-type or low-threshold voltage-activated calcium current), HCN2, Kv3.2, Kv4.2, and Kv1.1 are also found in Martinotti cells [60]. For the other described SST-interneuron subpopulations the molecular fingerprint is lacking however. But even so, the absence of CB [54] and the presence of CR [57] in the non-Martinotti cell populations may lead to different electrophysiological properties.

## 4. Connecting the Dots: SST-Interneurons in the General Cortical Connectivity Scheme and Functional Implications in Cortical Information Processing

### 4.1. Chemical Synapses

The discharge properties of individual interneurons ultimately depend on the network they are embedded in as interneurons and excitatory neurons are reciprocally connected [78]. Inhibitory synaptic connections are also widespread throughout the cortex, shaping networks within and between distinct interneuron subclasses [79, 80]. When dividing the interneurons in the three general nonoverlapping classes of PV-, SST-, and VIP- (5HT_3a_R-) interneurons, a consensual connectivity scheme has been proposed in which PV-interneurons somatically inhibit themselves. SST-interneurons do not inhibit each other, yet they do inhibit PV-interneurons. Lastly, VIP-interneurons strongly inhibit SST-interneurons, and to a lesser extent PV-interneurons [26, 81–83]. This simplified connectivity scheme is likely to show some variations across neocortical layers, in accordance with the laminar distribution and the presence of distinct interneuron subpopulations within these three groups.

In the following section, the input and output relationships of SST-subpopulations will be further reviewed as the heterogeneity in SST-interneuron populations in combination with lamina-specific distributions of their pre- and postsynaptically connected neurons suggests distinct functions in cortical processing. Several studies will be highlighted that look particularly into SST-subtypes, in agreement with the abovementioned subdivision of SST-interneurons.

### 4.2. The Input/Output Relationship of Distinct SST-Subpopulations

#### 4.2.1. Martinotti Cells

The best studied SST-interneurons, the Martinotti cells, have the highest connection probability with pyramidal cells, and the lowest with fast-spiking interneurons [27, 63, 73]. Firstly, Martinotti cells are involved in a disynaptic feedback inhibitory pathway of pyramidal neurons in layer V. Depending on the activity in the cortical column, the network switches between excitatory monosynaptic connections between neighboring layer V pyramidal neurons and a disynaptic inhibitory pathway between pyramidal neurons in layer V that excite Martinotti cells, which in their turn inhibit neighboring pyramidal neurons. The switch occurs when pyramidal neurons with low discharge rates mainly exciting each other start generating high frequency bursts that potently activate Martinotti cells through their facilitating nature. Subsequently, Martinotti cells exert their inhibitory activity mainly through dense inhibition onto layer I dendrites [84, 85], via fast GABA_A_ receptor-mediated synaptic input [27, 86]. Within these postsynaptic dendrites, Ca^2+^-spikes are generated, and prolonged regeneration of these spikes can elicit high frequency bursting in the soma of the pyramidal neurons in layer V [87]. When this activates layer V Martinotti cells, they can exert their negative feedback on the pyramidal neurons by inhibiting the sources of the Ca^2+^-spikes [61, 88–90]. The low-threshold spiking property of Martinotti cells indicates they can be activated by a small number of excitatory neurons [63] and since they are facilitating, they can be recruited by the repetitive firing of just one pyramidal neuron. Furthermore, the regulation of synaptic integration on excitatory dendritic tufts happens in a strictly compartmentalized manner, resulting in a highly focal inhibitory control of dendritic signaling. Since dendritic Ca^2+^-influx is an important factor in modifying synaptic transmission at glutamatergic synapses [91], Martinotti cell-mediated inhibition could act as a gate on synaptic plasticity, and this is on a spatial scale of individual glutamatergic inputs [92]. In the visual cortex, dendritic spikes enhance orientation selectivity of neuronal responses [93], suggesting an important regulating role for Martinotti cells on the output of their excitatory target cells residing in layer V. Altogether, this disynaptic feedback inhibitory mechanism is thought to be involved in preventing overexcitation within the cortical network. In addition, distal apical dendrites of pyramidal neurons in layer I receive long-range inputs from thalamus and other cortical areas that carry top-down feedback information required for cognitive processes and to filter out salient features of sensory inputs, vital in attention mechanisms. As such Martinotti cells dynamically modulate dendritic signaling to match the physiologically relevant input range [88, 94, 95]. It is in this sense that the Martinotti cell-mediated disynaptic feedback inhibition in layer V plays a pivotal role both in controlling local network processing and in long-range processing [27].

Martinotti cells present in layers II/III and V share much of the same properties, yet the laminar distribution of their presynaptic neurons suggests an additional, layer-specific level of control over these Martinotti cells. A study from Gentet et al. [96] investigating layer II/III Martinotti cells in the mouse barrel cortex shows that Martinotti cells can also inhibit layer I pyramidal cell dendrites tonically, whereas during active (or passive) whisking SST-interneurons hyperpolarize and relieve layer I dendrites from their inhibition to allow enhancement of excitatory inputs onto pyramidal neurons. The authors state that the cause of this hyperpolarization can be found in a lack of excitatory input on Martinotti cells common to other neurons in layer II/III, which is in contrast to layer V Martinotti cells that receive strong excitatory connections from neighboring pyramidal neurons. Furthermore, they suggest a stronger inhibitory input on these layer II/III SST-interneurons compared to the other layer II/III neurons. This inhibition likely comes to a large extent from VIP-interneurons, which are abundantly present in layer II/III but not so much in infragranular layers [26, 97, 98]. Considering the observation that layer I axons originate from higher cortical areas [99], this layer II/III disinhibitory mechanism relieving inhibition from pyramidal dendritic tufts under active conditions exerts another level of top-down control of sensory processing. As such this circuit can gate context-dependent processing and can integrate different streams of information in the neocortex [96]. A possible mechanism through which layer II/III SST-interneurons could exert these functions is through tonic release of GABA around synapses between layer II/III pyramidal neurons that express presynaptic GABA_B_-receptors on their glutamatergic boutons [100]. GABA_B_-mediated synaptic suppression can be rapidly and reversibly activated in a state-dependent manner. As such SST-interneurons can gate synaptic plasticity by relieving tonic inhibition from excitatory synapses when the interneurons become suppressed during sensory activity [101].

In addition to targeting layer I pyramidal dendrites, another study in rats reported layer II/III CB+ SST-interneurons targeting the axon initial segment of layer II/III pyramidal neurons, where action potentials are generated. This property has been previously attributed solely to Chandelier cells [102]. However, whereas Chandelier cells densely target axon initial segments [103], the number of SST-interneuron synapses is low. Perhaps these layer II/III targeting SST-interneurons belong to a distinct non-Martinotti cell subpopulation with medial extending axons that avoid layer I and remain in layer II/III, described by McGarry et al. [55], or to an as yet undescribed subpopulation (Figure 1, SST-interneuron 3). Since this study was performed in rats and not in mice, it is difficult to draw a consensus about the exact SST-subpopulation, as species-dependent differences even among rodents are considerable. Further analysis has indicated that these SST-interneurons targeting the axon initial segment also target the dendrites and soma of the pyramidal neuron. Regardless of which exact SST-subpopulation these interneurons belong to, these observations implicate functional roles of SST-interneurons across the entire extent of pyramidal cell output, from the dendritic tree to the axon initial segment, allowing control over both sub- and suprathreshold activity of pyramidal neurons [102].

#### 4.2.2. X94-Cells

SST-interneuron mediated inhibition on PV-interneurons in both layers II/III and V [26] could establish a shift from somatic PV-interneuron-driven inhibition to dendritic SST-interneuron-driven inhibition. Martinotti cells however have been reported to mainly target excitatory neurons [27, 63, 73]. SST-interneurons that target fast-spiking PV-interneurons therefore likely belong to a different non-Martinotti SST-population.

Several studies have linked layer IV and V X94-neurons to a PV-interneuron targeting circuit; however these X94-neurons have so far only been reported to target PV-interneurons within the thalamorecipient layer IV. The difference between layer IV and V X94-neurons can be found in their input sources, be it cortical (for layer IV X94-neurons) or thalamocortical (for layer V X94-neurons) [73, 74].

*Layer IV X94-Cells.* The X94-SST-subpopulation present in layer IV innervates only neurons within this layer in contrast to layer I-targeting Martinotti cells. The main input sources originate within the cortex, in contrast to the layer IV fast-spiking PV-interneurons that mainly receive thalamic input [104, 105]. The X94-neurons mostly innervate these fast-spiking PV-interneurons, which in their turn innervate layer IV principal neurons. As such, the layer IV SST-subpopulation is involved in a disinhibitory microcircuit within the thalamorecipient layer that could be involved in tuning the output of layer IV excitatory neurons and contribute to processing of sensory information [73].

*Layer V X94-Cells.* Layer V X94-cells have been reported to be involved in feedforward inhibitory mechanisms onto layer V pyramidal neurons. Generally, fast-spiking PV-interneurons have mainly been associated with thalamic feedforward inhibition [105], yet this only holds true under quiescent conditions. During active exploratory behavior, layer V barrel cortex SST-interneurons (labeled in the X94-mouse line) have been shown to undergo strong facilitation following high frequency thalamocortical input, whereas PV-interneuron activity rapidly depresses. This implies a delayed shift from somatic to dendritic inhibition during exploratory behavior. This SST-interneuron mediated dendritic inhibition leaves open a wider time window for synaptic integration and plasticity processes and maintaining the excitation/inhibition-balance within the cortical network [74].

#### 4.2.3. GIN-Cells

The faster kinetics reported in part of the GIN-labeled subpopulations [56] allows a more faithful propagation of distal inputs to the soma, imposing a better temporal segregation of inputs, in comparison to the remainder of GIN-neurons with slower kinetics and which probably consist of Martinotti cells (Figure 1, SST-interneuron 1). The slower kinetics may allow maximized temporal summation of excitatory postsynaptic currents, allowing more readily activation by bursting presynaptic pyramidal neurons [27, 56, 67].

#### 4.2.4. Long-Range SST-Neurons

The function of inhibitory long-range corticocortical projecting SST-neurons is only poorly understood and poses more difficulties to investigate due to their very low abundance. However, an interesting potential function is the communication and synchronization of activity within and between cortical areas through regulating rhythmic oscillations [41]. As such they could be important coordinators of perception, consciousness, or working memory mechanisms [106]. A role of locally projecting PV-interneurons has already been reported to generate and synchronize gamma-oscillations in the cortex [107], as well as locally projecting SST-interneurons in regulating cortical slow delta-oscillations [108] and theta-oscillations [109]. Further work is required however to investigate how long-range GABAergic neurons are connected within cortical networks to mediate synchronization of oscillatory activity in the adult mammalian cortex [41, 110]. Perhaps they could even regulate cross-modal interactions between cortical areas by forming bridges between sensory modalities.

### 4.3. Electrical Coupling

In addition to chemical synapses, SST-interneurons, like VIP- and PV-interneurons [111–113], are also electrically connected within their own subpopulations through gap junctions, or intracellular transmembrane channels [79, 114]. These gap junctions allow the direct passage of ions and molecules smaller than 10 kDa, mediating the bidirectional coupling of metabolic and electrical activities. This is typically an inhibitory feature as electrical coupling between pyramidal neurons is extremely rare [115–117]. Gap junctions between inhibitory neurons are formed by Connexin30.2 and Connexin36 protein subunits [118–120]. Particularly Connexin36 is crucial for electrical coupling and this protein has been found to overlap partially with SST-interneurons [121]. Within the SST-interneurons, evidence for gap junctions has been found for at least the subpopulation of Martinotti cells, in rat [122] and mouse [109] neocortex.

Even though gap junctions constitute a simpler form of signal transmission reminiscent of invertebrates, they are very important also during mammalian development when chemical synapses are still being established (for a review see [123]). However, gap junctions remain present in adult mammals and play an important role in network synchronization by coupling the membrane potential of connected cells. This leads to an increased probability of synchronized action potentials within inhibitory neurons, subsequently synchronizing the activity of other cortical cell populations and promoting oscillatory rhythmic activity and coincidence detection [117, 121].

### 4.4. Cholinergic Modulation of SST-Interneurons

Another line of evidence adding to the importance of interneurons and SST-interneurons in particular in cortical processes underlying mechanisms for attention, learning and memory, and cortical plasticity is the fact that SST-interneurons can be effectively depolarized by ACh via muscarinic [124] and nicotinic [125] receptor mediated mechanisms. An accumulating body of evidence suggests that this cholinergic neuromodulation is associated with learning-induced cortical plasticity processes [45, 76, 109, 126, 127]. In particular evidence has been found for layer II/III Martinotti cells and layer IV X94-SST-interneurons to carry cholinergic receptors. This adds another level of regulation to interneuron-mediated information processing within the thalamorecipient layer, as arousal and attention could as such control the entry of sensory information within this layer [73]. Furthermore, VIP-interneurons are also highly sensitive to cholinergic modulation [128, 129], adding to the neuromodulatory effect on layer II/III SST-interneurons and the downstream disinhibitory effects on cortical processing.

In summary, SST-interneurons maintain the excitation/inhibition balance within cortical networks by virtue of their central position in the connectivity schemes with both excitatory and inhibitory neurons. They mediate both feedback and feedforward inhibition to filter through relevant signals during active behavior and attention; they contribute to the switch from somatic to dendritic inhibition, opening windows of opportunity for integration mechanisms, and are involved in mediating rhythmic oscillations within the cortex. As such, the wide range of inputs and outputs of different SST-populations makes them quite diverse yet pivotal players at all levels of cortical network processing.

## 5. Implications of SST-Interneurons in Experience-Dependent Cortical Plasticity

It has long been appreciated that inhibition plays a key role in mediating different aspects of experience-dependent synaptic modifications in the brain to optimally process and respond to the world around us [16, 18, 130]. But how different interneuron cell types contribute to the underlying mechanisms and what the role may be specifically laid out for SST-interneurons remain enigmatic.

In this section several studies will be highlighted each putting forward SST-interneurons as interesting candidates in mediating cortical experience-dependent and learning-induced plasticity by virtue of their distinct laminar distribution and intrinsic and network properties.

### 5.1. Implications of SST-Interneurons in Learning-Induced Plasticity

Learning-induced plastic reorganization of cortical activity involves modifications and strengthening of excitatory synaptic inputs onto pyramidal neurons [131]. Such changes need to be balanced by a proportional amount of inhibition to prevent overexcitation and uncontrolled strengthening of synaptic connectivity, which could lead to impaired memory storage [132–134]. Multiple studies have already pointed towards a contribution of interneurons in learning-induced plasticity mechanisms as they observed increased GABAergic signaling through increased presynaptic GABA concentrations, upregulation of GABA_A_ receptor *α*1 subunit in postsynaptic terminals, and increased frequency of spontaneous inhibitory postsynaptic currents in the trained cortical areas following associative learning [135–137]. Additional studies looking closer into distinct inhibitory cell types found a specific involvement for SST-interneurons in learning-induced plasticity in several cortical areas such as auditory cortex [127], somatosensory cortex [138], or motor cortex [139].

In motor cortex, for example, SST-interneurons regulate the spatiotemporal specificity of learning-induced structural plasticity of excitatory synapses and thus acquisition of motor skills. Chen et al. [139] observed a learning-related spine reorganization that is typically restricted to layer I distal dendrites of layer II/III excitatory neurons. During motor training, a decrease in SST-interneuron boutons results in more depolarized layer I distal dendrites, which is in favor of synaptic potentiation and stabilization of learning-related spines. This is a property typically attributed to Martinotti cells, highlighting their importance in learning-induced plasticity. Furthermore, both optogenetic activation and suppression of SST-interneuron activity resulted in decreased spine reorganization and motor learning, implying that spine stability on distal dendrites is highly sensitive to a balanced amount of SST-interneuron mediated inhibition [139]. To add to this, in aging mammals, a decrease in GABAergic inhibition is associated with a decline in learning and memory abilities. Boosting GABAergic activity in these animals, and interestingly SST-interneuron activity, could counteract this age-related decline in cognitive functions [127, 140]. At the same time, PV-interneurons showed an increased number of boutons during learning, which is most likely a homeostatic response to the increased excitability of pyramidal neurons [139, 141].

An interesting mechanistic link through which SST-interneurons may be engaged in learning-induced plastic processes is through cholinergic signaling. The cholinergic system itself is already linked to learning processes and its associated cortical plasticity, as boosting the cholinergic system and stimulating the basal forebrain from which the cholinergic neurons project to the cortex, has an effect on learning and cortical information processing [142–146]. A link to show that cholinergic neuromodulation influences SST-interneuron activity in learning-related processes can be found in the observation that, in aged animals, both training and cholinergic enhancement by administering a cholinesterase inhibitor increase the number of SST-interneurons in the trained cortex [127].

Cholinergic receptors so far have been described in a subpopulation of SST-interneurons, more specifically layer II/III Martinotti cells and layer IV X94-SST-interneurons. Indeed, the study of Cybulska-Klosowicz et al. [138] suggested an involvement of layer IV SST-interneurons in the observed learning-induced plasticity processes following classical conditioning in the barrel cortex, as their densities increased as measured by means of activity-dependent upregulation of somatostatin in these cells [147]. They only observed an increased density of layer IV SST-interneurons. Increased activation of these interneurons by cholinergic activation could as such disinhibit thalamic inputs through inhibition of fast-spiking PV-interneurons [54, 73, 148]. These results seem to contrast with the reduced inhibition of SST-interneurons onto layer I-dendritic spines during motor learning described by the study of Chen et al. [139], but whereas they looked into layer II/III Martinotti cells, Cybulska-Klosowicz et al. [138] looked at layer IV SST-interneurons similar to neurons described in the X94-transgenic mice [54]. Together, these studies indicate that distinct SST-interneuron populations exert their function in contrasting ways to support learning-induced plasticity processes. Hence, fully understanding SST-interneurons will require specific methods allowing us to separate their distinct contributions to cortical network processing.

### 5.2. Plasticity in the Sensory Deprived Brain: SST-Interneurons in Ocular Dominance Plasticity

#### 5.2.1. SST-Interneuron Maturation during the Critical Period for Ocular Dominance Plasticity

Early in life, brain plasticity is enhanced during well-defined windows of brain development, or critical periods (CP), when experience-driven activity strongly modifies both structurally and functionally the neuronal basis originally laid out by a genetic blueprint [149, 150]. During these sensitive periods our brains are most malleable by outside experiences to allow us to optimally learn new skills that become “hard-wired” and stabilized, allowing us to benefit from these learned skills throughout life. Failure of a given sensory system during its particular critical period will cause the emergence of alternative brain organization patterns. A particularly well-studied example in the visual cortex is the critical period for ocular dominance plasticity (ODP), as pioneered by Hubel et al. [151]. OD is defined as the relative response of a neuron in the binocular primary visual cortex (V1b) to visual stimuli presented in one eye versus the other. When mammals are unilaterally visually deprived by eye closure during this CP, the balance of the responses to the two eyes shifts to the nondeprived eye, as it will take over cells in V1b originally responding to the contralateral deprived eye. Monocular deprivation in young animals is accompanied by the structural reorganization of both thalamocortical and corticocortical projections [152]. In addition, increased spine motility causes the destabilization of functional connections and is followed by spine loss on apical dendrites of layer II/III pyramidal neurons [153]. This parallels the reduction of cortical responsiveness to the deprived eye stimulation and later on this process is followed by the sprouting of new connections resulting in the strengthening of open eye inputs. As Martinotti cells are important input sources to these apical dendrites, this indicates a possible contribution of this inhibitory circuit or its maturation during the CP. After closure of the CP for OD, this shift will no longer occur so swiftly or will at least be much less effective [151, 154, 155]. Maturation of inhibitory neurotransmission is instructive for the timing of the CP. A certain threshold of inhibition is necessary to create a permissive environment for the detection of temporal activity differences between inputs from both eyes at the onset of the CP, whereas further maturation of inhibition results in a higher threshold that specifies the end of the CP and constrains ODP, for example, by reducing the capacity for long-term potentiation of cortical synapses [130, 156]. Interestingly, by changing the levels of inhibition, both the onset and closure of the CP for ODP can be shifted in time. More specifically, increasing the level of inhibition can be established by administering benzodiazepines [157] or removal of polysialic acid [158]. When this increase of inhibition is established before the CP would normally start, the inhibitory threshold to open the CP is reached earlier, resulting in a precocious induction of ODP. Subsequently, inhibition would normally mature to reach a second threshold that results in CP closure. However, when the level of inhibition is decreased (to lower levels, but still permissive for ODP), for example, by manipulations such as dark rearing that block inhibitory maturation, the CP closure will be delayed [17].

The role of PV-interneurons in this phenomenon of ODP has been the focus of many studies, as it has been long held that their functional maturation is crucial and even exclusive in opening the CP. PV-interneurons presumably also play an important role in closing it, although other mechanisms, such as epigenetic regulation of transcription or the maturation of the extracellular matrix to structurally stabilize the neural circuits, are also ascribed to CP closure [25]. Nevertheless, PV-interneurons are most likely only one part of the puzzle and recently the focus is starting to shift towards SST-interneurons as possible additional regulators of cortical plasticity. Like other GABAergic interneurons, SST-interneurons have a delayed postnatal maturation profile [159, 160]. Furthermore, the dendrite-targeting property of Martinotti cells controls synaptic integration, dendritic Ca^2+^-spikes, learning, and plasticity in general. As ODP requires structural rewiring of excitatory synapses onto dendritic spines of pyramidal neurons, SST-interneuron-mediated inhibition may be ideally localized within the cortical network to contribute to these processes. One study initiated the characterization of how SST-interneurons, and more specifically Martinotti cells in the GIN-mouse line, functionally mature during development and whether this can be correlated with the CP for ODP [161]. The authors showed that whereas PV-interneurons mostly mature early in the CP by receiving their fast-spiking behavior, allowing detection and transmission of precise spiking patterns in V1b necessary for the onset of ODP, SST-interneuron maturation involves an increased excitability and the gaining of slower membrane properties that arise not at the onset of the CP but progress in parallel with the CP as it reaches its peak. If this indeed involves Martinotti cells, their maturation will result in a stronger engagement of dendritic inhibition during the CP and will increase the time window over which Martinotti cells can integrate, respond to, and control competing synaptic inputs on the dendritic trees of pyramidal neurons.

Building further on this link between the maturation profile of SST-interneurons and ODP, another study transplanted GABAergic precursors for PV- and SST-interneurons from the medial ganglionic eminence (MGE) into cortical regions near V1b in 7-day-old mice. Thirty-three to 35 days after the transplantation, when the transplanted cells were at an equivalent age of postnatal days 26–28 (the age where the peak of the normal CP is situated in mice), this new wave of PV- and SST-interneuron maturation was found to induce a second window for ODP following short-term monocular deprivation, as measured by changes in the ocular dominance index. Remarkably, transplants depleted of PV-precursors but still containing SST-precursors were capable of inducing plasticity similarly robust to transplants that only contained PV-precursors but that were depleted in SST-precursors. Furthermore, transplants depleted in both cell populations could not induce ODP, indicating a mediating role for both PV- and SST-interneurons but not other MGE-derived cell populations. This study showed for the first time that, in addition to PV-interneurons, also SST-interneurons and more importantly their maturation profiles are crucial mediators for starting up windows of plasticity during development [162]. Despite the fact that this study does not distinguish between distinct SST-subpopulations originating from the MGE [163] and can therefore not specify whether different subsets of SST-interneurons have a differential effect on ODP, it is an important incentive to intensify future research into how SST-interneuron subtypes and their maturation profiles each may contribute to the mechanisms that support developmental plasticity.

#### 5.2.2. Adult Critical Period-Like Plasticity and SST-Interneurons

Critical periods of enhanced sensitivity are closed or severely constrained in adulthood; still certain manipulations that reduce the inhibitory level in V1b to a juvenile or CP-like condition of immature inhibition can cause the reopening of critical periods, allowing CP-like plasticity to occur well into adulthood [164–166]. This can be established by pharmacological reduction of intracortical inhibition, for example, by fluoxetine treatment, and insulin-like growth factor 1 [167] or 3-mercaptopropionic acid [168] administration, or by physiological manipulations such as environmental enrichment [169], food restriction [170], or short-term dark exposure [171]. The high level of spine turnover typically seen in young animals however drastically declines in adulthood [172]. As such, the loss of spines and thus the weakening of the deprived eye input are generally not occurring in adult animals exposed to monocular deprivation [173]. Since molecular brakes are already in place that block elaborate structural changes, also the thalamocortical as well as corticocortical reorganization becomes limited [152, 172]. As a result, the mechanisms controlling this type of unimodal plasticity in young versus adult animals differ, but both involve a crucial role for inhibitory transmission. However, manipulations to reinstate adult ODP do not specify exactly how the different cellular components in the inhibitory network (in addition to the extensively studied PV-interneurons) cooperate to reinduce ODP. Recently, the cortical disinhibitory VIP-SST-interneuron circuit was found to be involved in permitting adult ODP, as optogenetically increasing VIP-interneuron activity could rapidly increase V1 cortical responses following monocular deprivation [129, 174]. The authors could however not distinguish between different SST-subpopulations in this study, but considering the laminar distribution of VIP-neurons in layer II/III, the SST-population relevant in this circuit is likely restricted to this layer as well, putting Martinotti cells forward as good candidates.

Again, a link with cholinergic neuromodulation can be made as VIP-interneurons, as SST-interneurons receive direct nicotinic cholinergic inputs, which modulate the cortical state and sensory responses. As such, the VIP-SST disinhibitory circuit is well positioned to be a target for manipulation of experience-driven plasticity by this additional level of neuromodulation. It remains to be determined however how the cholinergic system converging on VIP-interneurons can be regulated. Recently, such a regulatory system has been found specifically in PV- and SST-interneurons, but not in VIP-interneurons. PV- and SST-interneurons both express Lynx family members of nAChR modulators that regulate the cholinergic signaling on these interneuron cell types. Specifically in PV-interneurons, Lynx1 functions as a cholinergic brake and has been observed to restrict adult ODP. Deletion of Lynx1 resulted in an enhanced nAChR signaling and successfully restored adult ODP [175]. In infragranular layer V and VI subsets of SST-interneurons, expression of Lypd6 modulates nAChR function by enhancing Ca^2+^-currents through these ion channels [75, 76]. It is not clear to what subpopulation they belong, let alone how this subpopulation may contribute to ODP, but Lypd6 is an interesting candidate that can mediate any potential influence of SST-interneurons onto cortical plasticity.

Additional evidence for an instructive role for SST-interneurons in adult ODP can be found in the structural plasticity of inhibitory synapses in ODP. Indeed it was found that adult monocular deprivation results in a loss of inhibitory synapses on distal apical dendrites of layer II/III pyramidal neurons [176], again hinting towards a contribution of dendrite-targeting SST-interneurons residing in layer II/III that receive input from VIP-interneurons. Furthermore, the changes in inhibitory synapses occurring here are not likely to only reflect a homeostatic mechanism to counteract a reduction in input, as a loss of synapses is seen after both monocular deprivation and restoration of vision. Indeed, SST-interneurons could so far not be found to be involved in homeostatic plasticity, in contrast to PV-interneurons [141]. More likely, the decrease in inhibitory synapses reflects a significant increase in thalamic inputs to layer II/III pyramidal neurons. As such, these plasticity mechanisms utilize a preexisting wiring scheme, leaving effective communication with other brain areas intact.

### 5.3. SST-Interneurons as Potential Integrators during Cross-Modal Plasticity

Extensive sensory loss results in a compensatory response of the spared senses, not only within the same modality (unimodal plasticity), but also between other spared modalities (cross-modal plasticity). This cross-modal plasticity especially occurs in multimodal areas, but, for example, also in monocularly driven visual areas, in contrast to ODP, which is mainly restricted to V1b [18, 177]. In cross-modal plasticity, the sprouting or unmasking of connections from spared modalities into the deafferented area can lead to functional recovery. However, the associated structural and functional changes are different during development compared to adulthood, underscoring that the way in which cross-modal plasticity manifests itself is (as in ODP) age-dependent [171, 178–180].

#### 5.3.1. SST-Interneurons in Developmental Cross-Modal Plasticity

In early deprived mammals, the plastic reorganization usually encompasses cortical, but also subcortical structural rewiring. A famous example consists of studies done on the congenitally blind mole rat, in which it was shown that the thalamocortical visual pathway is invaded by auditory input [181]. Also in mice subjected to congenital anophthalmia or neonatal enucleation, thalamic afferents can invade heteromodal thalamic targets [182]. But cross-modal plasticity does not only imply the structural and functional recruitment of the deprived cortical area by the intact senses to adapt to the sensory loss; also the intact senses themselves acquire enhanced processing of their modality specific input [183–185] and the underlying principles between these two adaptations differ [186]. It is evident that inhibitory neurotransmission is also a key factor in controlling both these cross-modal plasticity mechanisms. Several studies investigating early deprivation models have observed effects in GABAergic interneurons following loss of function in sensory modalities (for a review, see [179]). One implication for a role specifically for SST-interneurons during developmental cross-modal plasticity comes from early studies conducting monocular enucleation in young rats [187]. Monocular enucleation, or the surgical removal of one eye, is a more drastic deprivation paradigm than the eye suturing technique that is often used in ODP studies, as low contrast vision through the sutures and spontaneous retinal activity are completely absent (for a review see [188]). In early enucleated animals extensive structural remodeling takes place both in subcortical and in cortical structures [189] and a study of Jeffery and Parnavelas [187] showed an asymmetric distribution of SST-interneurons in these early enucleates. In the visual cortex contralateral to the removed eye, a slight increase in the density of SST-interneurons could be observed. No change was measured in other interneuronal cell markers, nor was the SST-interneuron asymmetry seen in adult long-term enucleated rats. These findings hint towards a link between SST-interneurons and the development of the input of terminals coming in from subcortical structures such as the dorsal lateral geniculate nucleus, as early enucleation often leads to extensive structural subcortical rewiring where other sensory systems can impinge on subcortical structures not normally assigned to them [181, 182]. This study was however only able to label layer II/III neurons, so possible changes occurring in deep cortical layers were not detected, although it can be hypothesized that deep SST-interneurons also play a role in these mechanisms as layer V X94-SST-interneurons receive thalamocortical inputs [73] and as such, by targeting PV-interneurons, may control inhibitory signaling onto thalamocortical connections, thereby restoring an altered excitatory balance caused by newly formed thalamocortical connections.

In another study in hamsters enucleated at birth, a reduction was seen in the number of CB-interneurons in layer V of V1, together with PV-interneurons, which additionally showed an increase in layer IV. It could be that the affected primary visual cortex adopts the GABAergic features of the auditory cortex through cross-modal rewiring [190]. The authors hypothesize changes in an alternate pathway for cortico- (thalamo-) cortical communication between V1 and neighboring-associated areas through layer V pyramidal neurons [191]. In mice, CB overlaps with SST-interneurons of the Martinotti cell-type, but whether this subpopulation is comparable to that in hamsters is unclear. Further studies will be required to elucidate whether and how SST-interneurons, or their maturation profile, can contribute in developmental cross-modal takeover of the deprived sensory areas.

In addition to reorganization within the deprived cortical area, loss of sensory function also leads to structural and functional alterations in the other intact modalities. In this context, one study looked into the barrel cortex of young mice that were olfaction-deprived starting from postnatal day 12. The authors described that olfactory deprivation recruits more GABAergic neurons in the barrel cortex. Specifically, using the GIN-mouse strain, they observed an increase in the number, the fine processes, and the encoding capacity for action potentials in SST-interneurons in the barrel cortex 1 week after olfactory deprivation [192]. It remains to be determined however in which layers such upregulation of SST-interneurons occurs, but the fact that they used GIN-labeled cells increases the possibility that they were looking at layer II/III SST-interneurons. As these SST-interneurons coordinate the activity of large populations of excitatory neurons, they could contribute to the regulation of intracortical layer II/III excitatory synapses.

#### 5.3.2. Adult Cross-Modal Plasticity

Cross-modal plasticity is not necessarily restricted to critical periods [193], but can also readily occur in adulthood as is observed in several species (cats: [194–196]; mice: [177, 180, 197]; rats: [198]; primates: [199, 200]; humans: [201–203]). Plastic changes occurring in adulthood are generally less profound and elaborate as in the young, as vast structural remodeling, especially involving subcortical pathways, is more limited due to maturation of structural brakes, but silent corticocortical connections could become functionally unmasked [204–206]. An indicative example for this hypothesis is that blindfolding of normally sighted humans could induce cross-modal activation of the deprived visual cortex on such fast time scales that it is unlikely to be mediated by the formation of new connections [207]. Adding importance to these preexisting corticocortical circuits in cross-modal plasticity, is the notion that primary sensory cortices are not* per se* unimodal, but are interconnected by direct reciprocal cross-modal corticocortical connections [208–213]. As such they can integrate multimodal inputs to adjust their output [208, 214]. It remains to be determined whether this involves direct corticocortical connections between primary sensory areas, or connections passing through secondary, possibly multimodal, sensory areas. For example, direct somatosensory inputs to the monocular visual cortex in rodents have been identified [177, 215, 216] as well as connections between the lateral extrastriate visual cortex and the temporal cortex [209, 217–221], but also direct long-range connections between primary visual, auditory, and somatosensory cortex [222]. Layer II/III excitatory neurons involved in intracortical and feedback connections are interesting substrates as they can integrate bottom-up and top-down sensory inputs [223].

Cross-modal modulation of neuronal responsiveness is mostly suppressive, which is indicative for an important role for inhibition in communication between different (early) sensory modalities [224–226]. Indeed, in V1, activation of auditory or somatosensory cortex leads to recruitment of infragranular inhibitory circuits through corticocortical connections that subsequently reduce layer II/III pyramidal activity. As such, distracting stimuli can be filtered out, leaving only the most salient sensory inputs [208]. It is also observed that loss of a sensory drive often leads to a homeostatic decrease in inhibition to make cortical neurons more responsive to remaining inputs [227] but is often followed by an increase exceeding normal inhibitory levels, possibly to counteract synchronous hyperexcitability [228, 229]. However, as homeostatic matching of inhibition to excitation is mainly ascribed to PV-interneurons [141], it is not clear how other distinct inhibitory subclasses behave and cooperate to permit or restrict cross-modal adaptations. An interesting candidate in these mechanisms is the SST-population of Martinotti cells as they reside in deep layers but can target supragranular excitatory neurons through their long ascending axon collaterals [27, 63, 88]. Furthermore, their low-threshold spiking behavior allows them to be activated by small numbers of layer V pyramidal neurons, which is in agreement with the low numbers of layer V excitatory neurons within V1 that could be activated by cross-modal inputs [208, 212]. This cross-modal modulation normally remains subthreshold, as suprathreshold responses are rare in early sensory areas, but following sensory deprivation perhaps these multisensory mechanisms can become gradually reinforced to allow stronger responsiveness to the intact senses, allowing the brain to make use of the already existing wiring pattern to recover sensory function. As such, SST-interneurons involved in multisensory integration [230] could be an interesting substrate during cross-modal plastic reorganization following loss of a sensory modality. It is possible that the VIP-SST-circuit is of particular importance here, as this circuit has already been found to be a potent modulator of sensory responses and to regulate cortical states by integrating long-range inputs from other brain regions [82, 129, 231, 232]. Again, considering the high sensitivity of VIP- and SST-interneurons for cholinergic signaling, it would be interesting to also consider neuromodulation in these mechanisms as this could possibly shift responses in multimodal neurons or areas [233].

Also in adult cross-modal plasticity both the takeover of the deprived cortical area and hypersensitivity of the spared modalities are observed [180]. Indeed, in adult mice following a brief period of visual deprivation, the sensory loss triggers potentiation of thalamocortical inputs into other primary sensory areas such as A1 [234]. This potentiation is probably not attributable to a stronger sensory drive, but to changes in feedforward and recurrent excitatory signaling within A1. Recently, a study in adult visually deprived mice has assigned a function for PV-interneurons in this strengthening of network processing within the intact sensory areas. PV-interneurons can establish a proportional inhibitory strength to match the increasing feedforward and recurrent excitatory drive within layers IV to II/III in the spared senses, which is a consequence of thalamocortical potentiation into layer IV [234]. PV-interneurons could this way increase spike precision and narrow tuning properties within the spared sense [186]. In contrast, intracortical processing within layer II/III was depressed and PV-interneurons did not have an effect within this layer. Still, the authors described an increase in miniature inhibitory postsynaptic currents in this layer [186]. Perhaps the VIP-SST-circuit is differentially recruited within this layer and could thus play an important role in response to altered sensory experience. Within the visual cortex itself, the exact opposite occurs as intracortical signaling is enforced at the expense of weaker thalamocortical inputs [186].

These studies indicate an interesting potential role for SST-interneurons in both the takeover of the deprived area by intact modalities and the hyperexcitability seen in the intact sensory areas, in both young and adult mammals. In any case, further cell-type specific studies are required to understand a possible contribution of SST-interneurons to these mechanisms.

## 6. Outlook

In summary, SST-interneurons make up a highly diverse group of interneurons that establish cortical, state-dependent inhibition at multiple levels and timescales in the cortical column. They are involved in both feedback and feedforward inhibitory and disinhibitory circuits and can as such integrate different streams of sensory information. Because of these properties, SST-interneurons are gaining more attention in plasticity research and indeed several lines of evidence suggest a central role for multiple SST-interneuron subtypes in regulating both learning- and sensory deprivation-induced plasticity phenomena, both during development and in adulthood. Particularly in layer II/III, where bottom-up and top-town sensory inputs integrate, SST-interneurons, and perhaps the VIP-SST-disinhibitory circuits, come forward as pivotal players in several plasticity mechanisms, suggesting interesting substrates for future research.

The use of transgenic mouse lines has proven to be an invaluable tool to characterize the distinct SST-interneuron subsets known today [54–56], and it will remain indispensable to further study the contribution of SST-subpopulations and their maturation profiles in cortical functioning and plasticity. Importantly, a thorough comparison on a molecular, morphological, and physiological level will remain essential to distinguish separate subtypes that can be compared between different studies. In addition, the ongoing progress of finding marker genes uniquely expressed in specific interneuron subsets [42] will certainly benefit the development of new and even more cell-specific neuroscience tools, for example, viral vector promoters for highly specific optogenetic or pharmacological cell targeting and manipulation strategies [235] to disentangle the causal links between subtype-specific interneuron function and plasticity.
